# Implementing focused echocardiography and AI-supported analysis in a population-based survey in Lesotho: implications for community-based cardiovascular disease care models

**DOI:** 10.1038/s41440-023-01559-6

**Published:** 2024-01-16

**Authors:** Emmanuel Firima, Lucia Gonzalez, Molulela Manthabiseng, Matumaole Bane, Blaise Lukau, Bailah Leigh, Beat A. Kaufmann, Maja Weisser, Alain Amstutz, Jasper Tromp, Niklaus Daniel Labhardt, Thilo Burkard

**Affiliations:** 1 Division of Clinical Epidemiology, University Hospital Basel, Basel, Switzerland; 2 University of Basel, Basel, Switzerland; 3 Swiss Tropical and Public Health Institute, Basel, Switzerland; 4 SolidarMed, Maseru, Lesotho; 5 University of Sierra Leone, Freetown, Sierra Leone; 6 Department of Cardiology, University Hospital Basel, Basel, Switzerland; 7 Division of Infectious Diseases and Hospital Epidemiology, University Hospital, Basel, Switzerland; 8 Ifakara Health Institute, Ifakara, Tanzania; 9 Saw Swee Hock School of Public Health, National University of Singapore, Singapore, Singapore; 10 Duke-NUS Medical School, Singapore, Singapore; 11 Medical Outpatient Department and Hypertension Clinic, ESH Hypertension Centre of Excellence, University Hospital Basel, Basel, Switzerland

**Keywords:** Focused echocardiography, Left ventricular hypertrophy, Community-based care, AI-supported analysis, Lesotho

## Abstract

In settings where access to expert echocardiography is limited, focused echocardiography, combined with artificial intelligence (AI)-supported analysis, may improve diagnosis and monitoring of left ventricular hypertrophy (LVH). Sixteen nurses/nurse-assistants without prior experience in echocardiography underwent a 2-day hands-on intensive training to learn how to assess parasternal long axis views (PLAX) using an inexpensive hand-held ultrasound device in Lesotho, Southern Africa. Loops were stored on a cloud-drive, analyzed using deep learning algorithms at the University Hospital Basel, and afterwards confirmed by a board-certified cardiologist. The nurses/nurse-assistants obtained 756 echocardiograms. Of the 754 uploaded image files, 628 (83.3%) were evaluable by deep learning algorithms. Of those, results of 514/628 (81.9%) were confirmed by a cardiologist. Of the 126 not evaluable by the AI algorithm, 46 (36.5%) were manually evaluable. Overall, 660 (87.5%) uploaded files were evaluable and confirmed. Following short-term training of nursing cadres, a high proportion of obtained PLAX was evaluable using AI-supported analysis. This could be a basis for AI- and telemedical support in hard-to-reach areas with minimal resources.

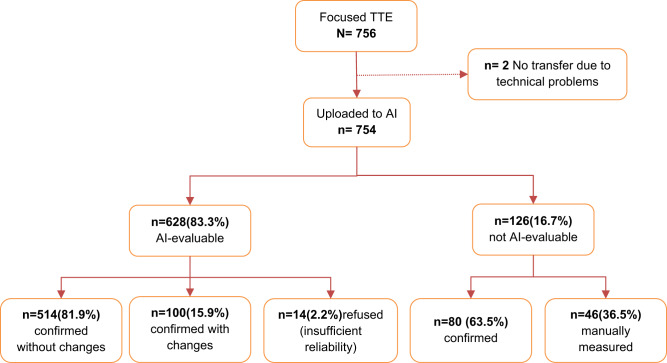

## Background

Left ventricular hypertrophy (LVH) is one of the most important predictors of morbidity and mortality in arterial hypertension [1]. As the prevalence of arterial hypertension increases, the burden of LVH is expected to rise [2]. The preferred modality for diagnosing and monitoring LVH is echocardiography, especially analysis of parasternal long axis views (PLAX) [3]. In low- and middle-income regions, such as sub-Saharan Africa, diagnosis of LVH is challenging due to limited ultrasound devices and trained staff [4]. In Lesotho, these challenges are particularly self-evident, with only 0.07 doctors per 1000 inhabitants and even fewer cardiologists or cardiology centers [5].

Point-of-care-ultrasound (POCUS) devices are cheap and practical alternatives to more expensive cart-based trans-thoracic ultrasound devices [6]. POCUS devices for focused cardiac imaging might improve the diagnosis of LVH in these regions, especially when combined with artificial intelligence (AI) techniques to analyse the acquired loops. Utilizing non-expert sonographers to operate POCUS, access to focused cardiac imaging can be improved. However, challenging aspects of using focused echocardiography include user training, data handling in remote areas, and analyzing and interpreting acquired images [7].

A recent clinical validation study of an automated deep learning workflow (Us2.ai, Singapore) demonstrated that deep learning-based interpretation of echocardiographic DICOM images is interchangeable with expert human sonographers [8]. Earlier studies conducted in Kenya [9] and Uganda [10], where novice trainees received 1–2 days hands-on training, showed mixed image quality and measurement results, compared to experts. In our study, we pragmatically implemented focused echocardiography within a population-based survey in Lesotho. Images were obtained by short-term trained nurses and nurse assistants using hand-held ultrasound devices, followed by AI-supported interpretation. The aim of the current analysis was to examine the proportion of evaluable echocardiograms obtained using this pragmatic approach. This analysis is premised on the fact that since the validated AI interpretation is comparable to human experts, the possibility for non-expert sonographers to obtain images that are evaluable by AI, could support LVH evaluation in settings where experts are lacking.

## Methods

This study is based on data obtained during a study on end-organ damage among individuals found to have elevated blood pressure that was nested within a population-based non-communicable disease survey in Butha Buthe and Mokhotlong districts in Lesotho. The survey was part of a community-based chronic care program in Lesotho (ComBaCaL) [11]. Lesotho’s National Health Research Ethics Committee (NH-REC) approved the study protocol (NH-REC ID 139-2021), and participants provided written informed consent.

A training manual was developed based on the 2015 Recommendations for Cardiac Chamber Quantification in Adults by the American Society of Echocardiography and the European Association of Cardiovascular Imaging, and the Quick Reference Guide from the ASE Workflow and Lab Management Task Force 2018 [7]. Sixteen nurses/nurse-assistants were selected to undergo a 2-day intensive hands-on training, followed by one week of practical on-field supervision. The training was given by an experienced physician and cardiac sonographer, and focused on the correct acquisition of parasternal long axis (PLAX) views, export of the loops as digital imaging and communication in medicine (DICOM) files, and external storage of the loops for later core lab analysis. See supplementary material for the timetable of the training, and the standard operating procedure for image acquisition. At the end of the training period, and before commencement of the study, the nurses/nurse assistants were assessed by the trainers, and showed ability to identify and obtain simple PLAX views, as well as store and export loops.

**Fig. 1:**
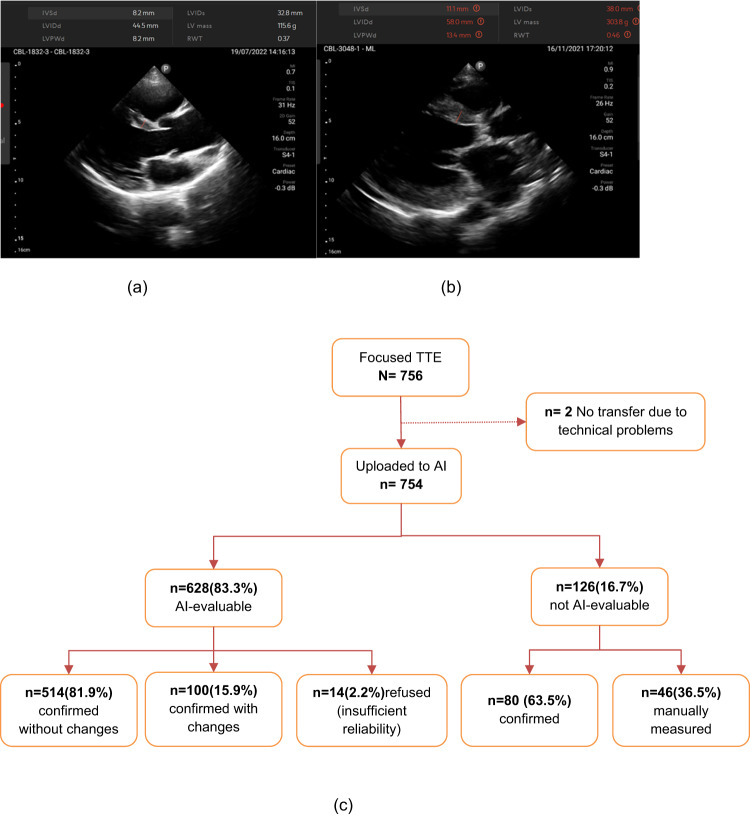
**a** AI analysis of normal left ventricle (**b**) AI analysis of enlarged left ventricle. **c** Study flow with results of AI analysis. TTE transthoracic echocardiography, AI artificial intelligence

Data collection continued from 2nd November 2021 to 30th November 2022. Research teams visited urban or rural areas and performed the focused echocardiography at participants’ homes (Fig. 1a, b). Philips Lumify Ultrasonography device [12] was used for the focused echocardiographic procedure. At least two loops of PLAX views lasting 3 s each were obtained and stored as DICOM files. These files were then synchronized to a cloud drive as soon as survey teams returned to an area with internet coverage.

DICOM files were downloaded onto an external drive, and analyzed using deep learning-powered software (Us2.ai, Singapore) [8] at University Hospital Basel (UHB). The deep learning-powered workflow automatically classifies and analyzes echocardiographic images and videos. For the current study, we measured ventricular dimensions including end-diastolic interventricular septal thickness, end-diastolic and end-systolic left ventricular internal diameter, and end-diastolic left ventricular posterior wall thickness.

Figure 1c shows the workflow of the current study. First, DICOM files were uploaded for automated analysis and reviewed by a trained investigator (physician). The investigator then classified results of the automated analysis as “confirmed without changes”, if results were fully acceptable, “confirmed with changes” if measurements were manually edited after automated measurement, or as “refused” if image quality was inadequate for reliable measurements.

Images judged as “not evaluable” by the AI algorithm were either “confirmed” by the first investigator as not measurable if images did not allow manual measurements. Otherwise, manual measurements were performed (“manually measured” on Fig. 1c). Finally, an experienced senior board-certified cardiologist (TB) reviewed and approved all measurements and group assignments of individual datasets.

The study’s main outcome was the proportion of evaluable PLAX views by the software. Evaluable measurements were expressed as percentages. Additionally, relying on the AI algorithm to correctly obtain the true cardiac dimensions, since it has been validated to be comparable to human experts, we sought to assess any effects on initial AI measurements where manual edits had been done. Thus, a secondary outcome included the difference in means (standard deviation [SD]) and mean absolute differences between the edited and non-edited deep learning results in the “confirmed with changes” category.

## Results

In total, 16 (13 [81% women]) nurses/nurse-assistants obtained focused echocardiographs from 756 participants. The participants’ median age was 56 years (IQR: 42–68), and their body mass index was 25.2 (IQR: 21.9–30.1) kg/m². All 756 unique DICOM files were downloadable at the UHB. Of these, 754 (99.7%) were successfully uploaded to AI platform.

Out of 754 files, 628 (83.3%) were evaluable by AI. Of these, 514 (81.9%) were confirmed by the independent cardiologist without changes, and 100/628 (15.9%) were confirmed with changes. In total, 660 (87.5%) of all uploaded files were evaluable either by AI-supported analysis (93%) or by additional manual measurement (7%). Of the 126 images not evaluated by AI, 46(36.5%) could be assessed manually. The main reason for the AI algorithm’s non-analysis was low quality of images and insufficient number of loops for high-confidence analysis (Fig. 1).

Table 1 shows that the mean (SD) and absolute differences between the unedited and manually edited results in the “confirmed with changes” category were without relevant differences.

**Table 1:** Differences in measurement before and after manual editing in the 100 AI-evaluated files that were confirmed with changes

Dimension	Mean (SD) before edit	Mean (SD) after edit	Mean difference (95% CI)	MAD
IVSd, mm	9.7 (2.0)	9.4(1.9)	0.3(0.1, 0.5)	0.40
LVIDd, mm	36.7(3.7)	37.8(3.7)	−1.1(−1.4, −0.8)	1.28
LVIDs^a^, mm	27.0(4.4)	27.0(4.4)	0	0
LVPWTd, mm	8.9(1.3)	8.8(1.0)	0.1(−0.03, 0.22)	0.09
LVM, g	102.2 (29.6)	102.8 (23.8)	−0.5(−4.1, 3.0)	9.16
RWT, mm	0.49(0.08)	0.47(0.06)	0.02(0.01, 0.03)	0.02

*MAD* mean absolute difference, *SD* standard deviation, *CI* confidence interval, *IVSd* end-diastolic interventricular septal thickness, *LVIDd* end-diastolic left ventricular internal diameter, *LVIDs* end-systolic left ventricular internal diameter, *LVPWTd* end-diastolic left ventricular posterior wall thickness, *LVM* left ventricular mass, *RWT* relative wall thickness

^a^no edits done

## Discussion

We demonstrate that nurses/nurse-assistants with limited training can assess LVH in a resource-limited setting using an inexpensive POCUS device with AI-powered interpretation. Our results underline the potential of non-physician healthcare workers to evaluate and monitor LVH in resource-limited settings. The combination of a POCUS device with pre-installed AI decision support tools for automated interpretation can enable cardiac screening and diagnosis without transferring acquired loops [13].

In this study, the nurses/nurse-assistants were trained to acquire only PLAX because this is easy to learn within a limited time, while offering sufficient information to characterize LVH, compared to other cardiac views. This resulted in most examinations being analysable by AI algorithms. Reassuringly, the pre-determined quality requirements of the software were stricter than those often used by trained cardiologists. Specifically, the software demanded more loops for high confidence analysis than the cardiologist, which explains why 46 studies could be manually read but not by the AI-software. A clear advantage of the software was its time-efficiency. Most automated measurements were only checked and confirmed, substantially reducing the required effort of manually performing all measurements. Importantly, in the 100 examinations that were “confirmed with changes”, differences before and after editing by the investigators were minimal, and majority of cases clinically irrelevant [14].

Our results suggest that an AI-supported POCUS exam by non-physician healthcare workers improves access to echocardiography. In two recent scoping reviews on community-based care for cardiovascular diseases in sub-Saharan Africa [15, 16], evidence was lacking concerning decentralization of health care delivery where a majority of care was delivered in patients’ households. Findings from the reviews, however, suggested high acceptability of community care among patients and service providers. In our study, the overall proportion of images confirmed without change to total uploaded data was 68% (514/754). However, among 628 images that were evaluable by AI, 81.9% (514) were confirmed without change. Among those accepted with changes, the impact of the expert was clinically negligible. Only in 14 (2.2%) of examinations, was the image quality deemed by the experts to be insufficient for measurement, which suggests a reassuringly high level of quality required by the AI. These numbers suggest the potential for nurses/nurse assistants in making echocardiography available in hard-to-reach settings. Therefore, scaling an AI-supported POCUS examination to diagnose cardiac disease by non-physician healthcare workers might be feasible and acceptable.

## Strength and limitations

A strength of our study is that among a team of healthcare workers with short training and AI-supported analysis, it is feasible to obtain LVH measurements in 87% of participants, despite limited training, hand-held sonography devices, difficult imaging conditions, and limited personal resources for analysis. Our results encourage further study of focused echocardiography and AI support for additional views and parameters like left ventricular ejection fraction, which could solve an unmet clinical need in hard-to-reach environments. Such study could explore user experiences to improve overall outcomes.

Due to the limited capacities in our setting, we could not carry out additional standard echocardiography by fully-trained cardiac sonographers using high-end devices as control. Thus, we could not determine if, under better conditions, results of the AI analysis could have been different for the 126 and 14 participants without AI analysis, or not confirmed AI analysis, respectively. Additionally, images could not be allocated to individual examiner. Thus, it is not clear if all nurses/nurse assistants performed well.

## Conclusion

Following short-term training of nurses/nurse assistants, a high proportion of obtained PLAX was interpretable using AI-supported analysis regarding left ventricular dimensions and parameters indicating LVH. This could be important for telemedicine support for cardiac imaging in hard-to-reach areas with minimal resources.

### Supplementary information

Supplementary information
