# Supplementary material for: Implementing focused echocardiography and AI-supported analysis in a population-based survey in Lesotho: implications for community-based cardiovascular disease care models
Source: Hypertens Res. 2024 Jan 16;47(3):708–13. doi: 10.1038/s41440-023-01559-6 (PMC10912015; doi:10.1038/s41440-023-01559-6)
Supplement: Supplementary file 1 — Supplementary information [file 41440_2023_1559_MOESM1_ESM.docx]

Appendix 1

Brief communication

Implementing focused echocardiography and AI-supported analysis in a population-based survey in Lesotho: Implications for community-based cardiovascular disease care models

**Description of the nurse training.**

Nurse training was done at the Mokhlotlong Hospital, Lesotho by a physician and experienced sonographer. Teaching focused on cardiac parasternal long axis view (PLAX) image acquisition using the use of the handheld device.

Time schedule of the training is shown in Table A1. Theoretical teaching included the topics of basic concepts of ultrasound, basics of ultrasound physics, artefacts, transducers, optimization of image quality (e.g. depth, gain), preparation of the study participant, tablet and probe, ideal positioning of the study participant, image acquisition of PLAX (placement of the probe, cardiac structures that need to be visualized) and pitfalls when acquiring PLAX.

Training materials were based on the 2015 Recommendations for Cardiac Chamber Quantification in Adults by the American Society of Echocardiography and the European Association of Cardiovascular Imaging, and the Quick Reference Guide from the ASE Workflow and Lab Management Task Force 2018.

After the training, nurses/nurse assistants received the study training materials including the guidelines, the training presentations and a standard operating procedure for guidance in the field. The standard operating procedure was for confidential internal use only – some figures are blanked in the context of this appendix.

Supplementary table 1 – Focused PLAX Training Timetable

| **Time** | **Modules** |
| --- | --- |
| **DAY 1** | |
| 09:00 AM – 09:15 AM | Welcoming remarks |
| 09:15 AM – 09:30 AM | Introduction |
| 09:30 AM – 10:30 AM | Basic ultrasonographic concepts |
| 10:30 AM – 11:00 AM | Technical issues ultrasonography |
| 11:00 AM – 11:15 AM | Break |
| 11:15 AM – 1:00 PM | Tips on how to acquire PLAX |
| 1:00 PM – 2:00 PM | Lunch Break |
| 2:00 PM – 3:00 PM | Pitfalls and quality check |
| 3:00 PM – 4:45 PM | Hands on training |
| 4:45 PM – 5:00 PM | Wrap up of the day |
| **DAY 2** | |
| 08:30 AM – 09:00 AM | Recap of Day 1 |
| 09:00 AM – 10:30 AM | Hands on training |
| 10:30 AM – 10:45 AM | Break |
| 10:45 AM – 1:00 PM | Hands on training |
| 1:00 PM – 2:00 PM | Lunch break |
| 2:00 PM – 4:45 PM | Hands on training |
| 4:45 PM – 5:00 PM | Q&A – Closing remarks |

**Standard operating procedure (SOP) PLAX acquisition:**

All recommendations and measurements are based on the 2015 Recommendations for Cardiac Chamber Quantification in Adults by the American Society of Echocardiography and the European Association of Cardiovascular Imaging, and the Quick Reference Guide from the ASE Workflow and Lab Management Task Force 2018. The SOP is based on the general and PLAX chapters of the SOP used in the CoArthHA trial (Identifying most effective treatment strategies to control arterial hypertension in sub-Saharan Africa – a randomized controlled trial).

**1.1 Preparation of the patient**

Patient lies on lef lateral side, left arm up. Examinator sits on the left side of the patient. Patient takes off the shirt

**1.2 Preparation of tablet and probe**

Switch on tablet, then choose lumify app.

1. Create patient,

2. Press start exam

3. Connect the probe to the tablet

4. Put Jelly on probe

Adjust gain, depth

To acquire loops, press ‘save loop’

**1.3 Image Acquisition**

**1.3.1 Parasternal Long Axis**

For acquisition of a parasternal long axis patient should be positioned on the left lateral side. The transducer has to be placed in the 3rd or 4th intercostal space on the left side right close to the sternum and the marker of the transducer (grey stripe on the side) points towards the right shoulder. Depending on the axis of the heart of the subject, the transducer has to be corrected with the aim that the ventricle is as long stretched as possible focusing on the mid-portion and base of the left ventricle. Usually the apex could not be seen in this view.


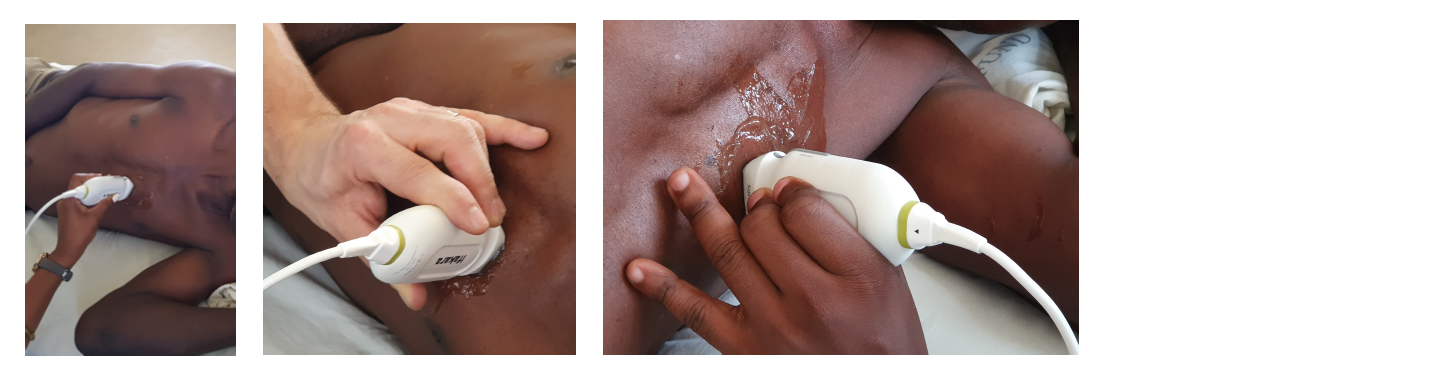


Transducer should be adjusted until the there is no (or minor) angle between the septum and the aortic sinus. The image should be focused on the basal segments of the left ventricle, the aortic valve and the mitral valve together with the left atrium. The aortic valve should close in a symmetric manner with the tips in the middle of the sinus of aortae.

Depth should be adapted, so that one centimeter is at the bottom to visualize structures posterior to the left ventricle.

Linear internal measurements of the LV should be performed in the parasternal long-axis view


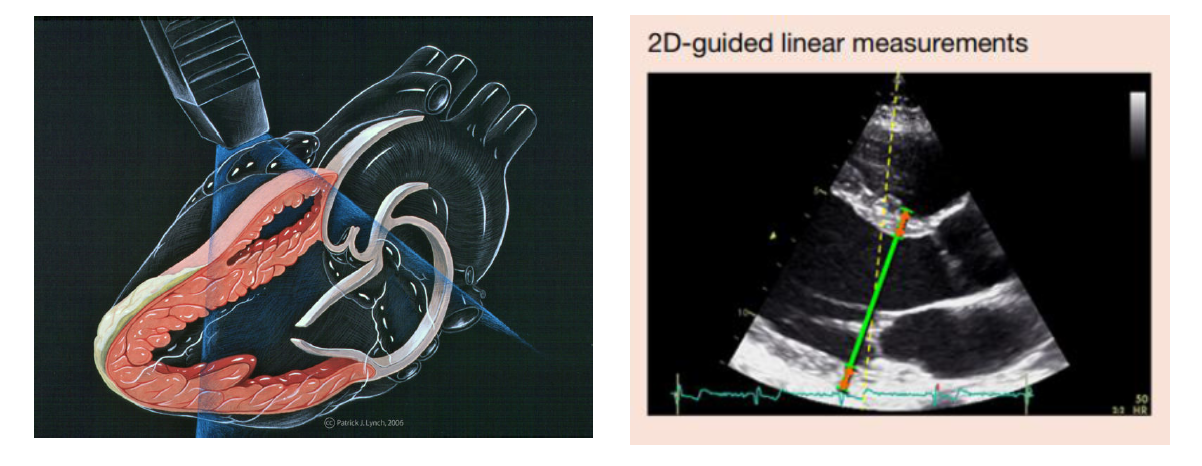


2 images illustrating position of the probe in relation to the ultrasound device and how to place measurements according to the guidelines

When the optimal position is found:

- **Aquire 2 loops, each with 3 heart cycles** (resp. 3 seconds)
